# *In vivo* analysis of ankle joint kinematics and ligament deformation of chronic ankle instability patients during level walking

**DOI:** 10.3389/fbioe.2024.1441005

**Published:** 2024-08-06

**Authors:** Yaokuan Ruan, Shengli Wang, Nan Zhang, Zhende Jiang, Nan Mei, Pu Li, Lei Ren, Zhihui Qian, Fei Chang

**Affiliations:** ^1^ Department of Orthopedics, The Second Hospital of Jilin University, Changchun, China; ^2^ Key Laboratory of Bionic Engineering (Ministry of Education, China), Jilin University, Changchun, China; ^3^ Department of Radiology, The Second Hospital of Jilin University, Changchun, China; ^4^ Orthopaedic Surgeon Department of Orthopaedic Surgery, Nara Medical University, Nara, Japan; ^5^ Health Technology College, Jilin Sport University, Changchun, China

**Keywords:** chronic ankle instability, lateral ligament, kinematics, biomechanics, ligament elongation, dynamic biplane radiography

## Abstract

**Introduction:** Chronic ankle instability (CAI) carries a high risk of progression to talar osteochondral lesions and post-traumatic osteoarthritis. It has been clinically hypothesized the progression is associated with abnormal joint motion and ligament elongation, but there is a lack of scientific evidence.

**Methods:** A total of 12 patients with CAI were assessed during level walking with the use of dynamic biplane radiography (DBR) which can reproduce the *in vivo* positions of each bone. We evaluated the uninjured and CAI side of the tibiotalar and subtalar joint for three-dimensional kinematics differences. Elongation of the anterior talofibular ligament (ATFL), calcaneofibular ligament (CFL), and posterior talofibular ligament (PTFL) were also calculated bilaterally.

**Results:** For patients with CAI, the dorsiflexion of the tibiotalar joint had reduced (21.73° ± 3.90° to 17.21° ± 4.35°), displacement of the talus increased (2.54 ± 0.64 mm to 3.12 ± 0.55 mm), and the inversion of subtalar joint increased (8.09° ± 2.21° to 11.80° ± 3.41°). Mean ATFL elongation was inversely related to mean dorsiflexion angle (CAI: rho = −0.82, *P* < 0.001; Control: rho = −0.92, *P* < 0.001), mean ATFL elongation was related to mean anterior translation (CAI: rho = 0.82, *P* < 0.001; Control: rho = 0.92, *P* < 0.001), mean CFL elongation was related to mean dorsiflexion angle (CAI: rho = 0.84, *P* < 0.001; Control: rho = 0.70, *P* < 0.001), and mean CFL elongation was inversely related to mean anterior translation (CAI: rho = −0.83, *P* < 0.001; Control: rho = −0.71, *P* < 0.001). Furthermore, ATFL elongation was significantly (CAI: rho = −0.82, *P* < 0.001; Control: rho = −0.78, *P* < 0.001) inversely correlated with CFL elongation.

**Discussion:** Patients with CAI have significant changes in joint kinematics relative to the contralateral side. Throughout the stance phase of walking, ATFL increases in length during plantarflexion and talar anterior translation whereas the elongation trend of CFL was the opposite. This understanding can inform the development of targeted therapeutic exercises aimed at balancing ligament tension during different phases of gait. The interrelationship between two ligaments is that when one ligament shortens, the other lengthens. The occurrence of CAI didn’t change this trend. Surgeons might consider positioning the ankle in a neutral sagittal plane to ensure optimal outcomes during ATFL and CFL repair.

## 1 Introduction

Ankle sprains are one of the most common injuries in sports and daily life (Soboroff et al., 1984). Up to 40% of patients who suffer from lateral ankle sprains develop long-term symptoms, such as frequent sensations of ankle instability and recurring sprains (Fong et al., 2007; Yu et al., 2023). These lingering symptoms are often referred to as chronic ankle instability (CAI) (Freeman, 1965; van Rijn et al., 2008). Clinical findings suggest that there is a correlation between CAI and degenerative changes in the ankle joint, which may increase the incidence of osteochondral lesions of the talus as well as osteoarthritis of the ankle joint (Deleu et al., 2021; Lalevée et al., 2023; Mousavi et al., 2023). Many researchers believe that abnormal joint motion and elongation of ligaments are significant factors contributing to the progression of osteoarthritis in patients with CAI (Caputo et al., 2009; Deleu et al., 2021; Ye et al., 2021).

Current research suggests that patients with CAI exhibit different kinematic outcomes compared to healthy persons, such as increased plantarflexion and inversion of the ankle joint (Balasukumaran et al., 2020a; Herb C. and Shank K., 2023; Mousavi et al., 2023). These changes in kinematics result in the ankle being positioned in a more open state, which decreases the mechanical stability of the joint and increases the risk of recurrent sprains (De Ridder et al., 2013; Li et al., 2019; Yu et al., 2024). To gain a deeper understanding of the phenomenon, numerous studies have utilized motion capture systems to reveal the kinematic differences between the ankle joints of patients with CAI and those of healthy individuals (Monaghan et al., 2006; Piming et al., 2023). However, this method considers the ankle joint as a whole and is unable to distinguish between the tibiotalar and subtalar joints (Wu et al., 2002; Lenz et al., 2020). Analyzing the tibiotalar and subtalar joints separately will help understand the kinematic changes within the patient’s joints and lead to a deeper understanding of the disease.

Kinematic changes also affect ligament deformation (Agostinone et al., 2021). In particular, the lateral collateral ligament, including the anterior talofibular ligament (ATFL), calcaneofibular ligament (CFL), and posterior talofibular ligament (PTFL), which are often injured clinically (Yu et al., 2022). Many researchers have employed methods such as cadaveric experiments or simulation analysis to study the impact of lateral collateral ligament injury on joint stability and the deformation of the ATFL, CFL, and PTFL (Nigg et al., 1990; Zhang et al., 2014; Chang et al., 2021; Boey et al., 2022). These studies qualitatively explored the correlation between joint motion and ligament elongation. However, there is limited *in vivo* data describing how the ligament functions to stabilize the ankle joint during dynamic loading activities. A more thorough comprehension of the *in vivo* role of ligaments in patients with CAI is necessary to enhance current treatment strategies and rehabilitation programs.

As a method of directly tracking the position of the skeleton, dynamic biplane radiography (DBR) overcomes the limitations of previous methods. The accuracy of DBR in measuring kinematics has been confirmed, with documented discrepancies of less than 1 mm and less than 1° (Wang et al., 2015; Kessler et al., 2019). Combined with MRI data, the DBR enables *in vivo* assessment of ligament deformation during dynamic activities (Englander et al., 2019; Chiba et al., 2021). A recent study used the DBR to analyze the ankle’s kinematics in seven key poses during the stance phase and obtained similar results (Cao et al., 2019). However, using fewer key poses may make it difficult to accurately represent the complete dynamic cycle of joint kinematics.

This study aimed to analyze the impact of CAI on tibiotalar and subtalar joint motion and determine ligament elongation during the stance phase of walking by DBR. The correlation between the elongation of the lateral collateral ligament and kinematics was examined during walking, as well as the correlation between the elongation of the ligaments. We hypothesize that patients with CAI will exhibit altered joint kinematics and ligament elongation patterns compared to healthy controls, which may contribute to the observed instability and increased injury risk. The results may deepen the understanding of the dynamic changes in ankle kinematics and ligament deformation for patients with CAI, and therefore supply inspiration for the corresponding clinical practice.

## 2 Materials and methods

### 2.1 Subject recruitment

This research was granted approval by the Ethics Committee of our hospital, and informed consent was obtained from all participants. The data were obtained from a convenience sample of 12 patients selected, with an average age (and standard deviation) of 35.4 ± 13.0 years and an average body mass index of 23.9 ± 3.1 kg/m^2^. The demographic characteristics of the recruited subjects are detailed in Supplementary Material (Supplementary Table S1).

The inclusion criteria for CAI patients were as follows: (1) an initial ankle sprain occurring more than 12 months prior; (2) recurrent ankle sprain (at least two sprains in the same ankle), episodes of giving way (more than twice in the recent 3 months); (3) positive results on the talar tilt test and anterior drawer test; (4) MRI reveals the presence of ATFL injury with or without PTFL and CFL injury; (5) Foot and Ankle Outcome Score (FAOS): <75% in 3 or more categories. Exclusion criteria in the study were: (1) fractures and surgical history of the hindfoot; (2) patients who had suffered acute trauma in either lower extremity within the past 1 month or had experienced a sprain less than 1 month prior; (3) those diagnosed with osteoarthritis in either lower extremity, or other medical conditions that could impact normal gait; (4) patients with poor balance, or at risk of falling when walking on the raised laboratory walkway.

### 2.2 DBR motion capture

**FIGURE 1:**
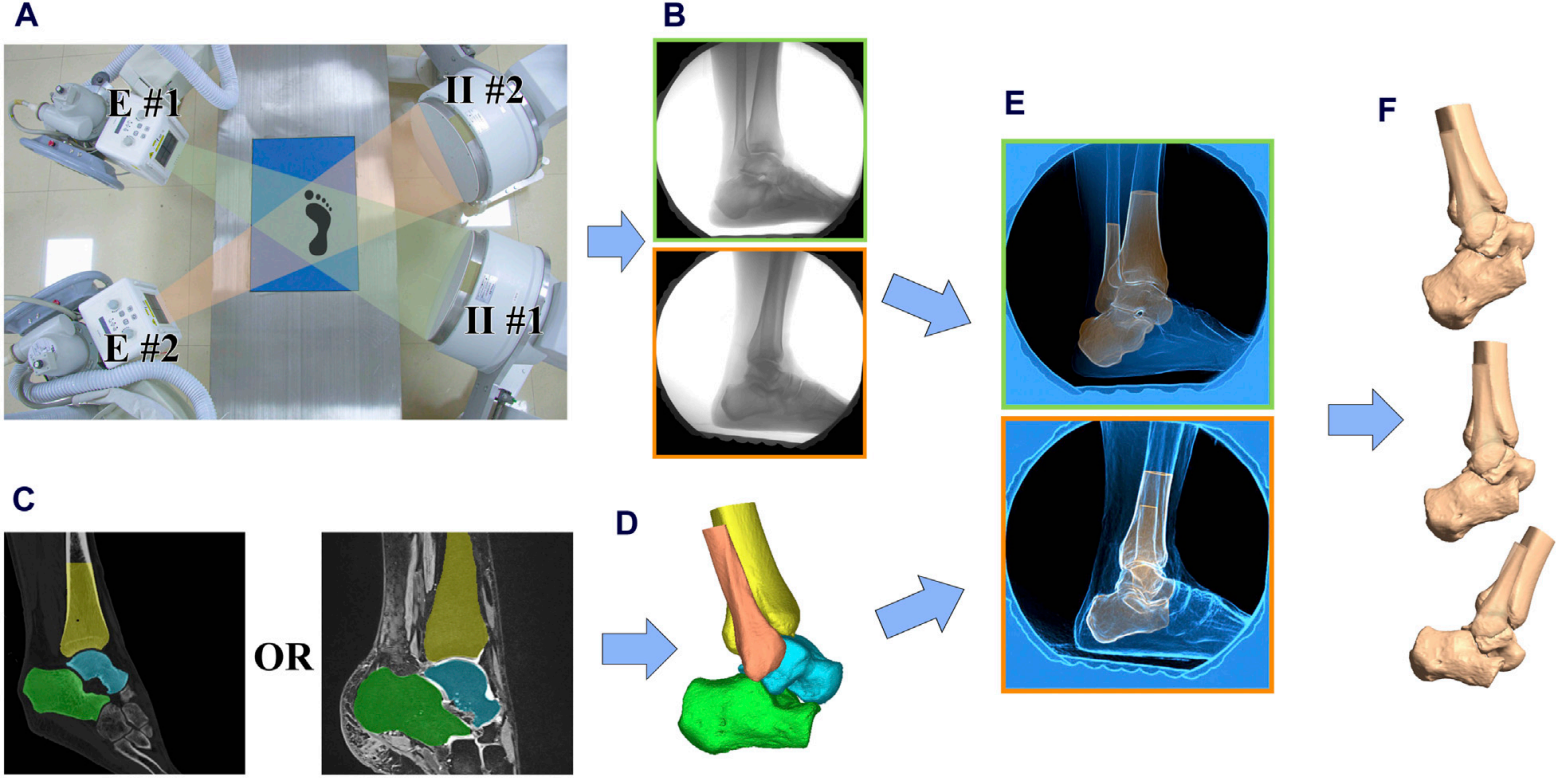
Data collection and processing workflow. **(A)** The image intensifiers (II #1 and II #2) processed images created by x-rays from the emitters (E #1 and E #2); **(B)** Participants performed 3–5 trials of walking at a self-selected pace within the biplane radiographic imaging system which collected at 120 Hz; **(C)** CT or 3D MR scans were collected and used to create subject-specific 3D bone models; **(D)** 3D model of the ankle joint with ligament origins and endpoints labeled; **(E)** 3D bone models were matched to the biplane radiographs using a validated process; **(F)** Relative translations, rotations and ligament elongation were calculated.

A previously employed DBR system (Imaging Systems and Service Inc., United States) was utilized to measure the kinematics of the tibiotalar and subtalar joints (Wang et al., 2023). Participants walked at a self-selected speed on a raised laboratory walkway. Three valid trials were recorded for each ankle (Figure 1A).

Biplane radiographs of the ankle were obtained for a duration of 1-2 s in order to capture the stance phase at a rate of 120 frames/s (imaging parameters: 54–56 kV, 80–100 mA, 0.9 ms pulse width) (Figure 1B).

### 2.3 Imaging data and model-based markerless tracking

Quantification of *in vivo* joint kinematics from DBR images requires three-dimensional (3D) reconstruction data for each bone. In order to reduce the radiation exposure of the patients, a 3 T magnetic resonance imaging (MRI) scanner (Prisma, Siemens Medical, Solutions United States) was used to reconstruct bone models in three dimensions. Sagittal, coronal, and axial images were obtained from the participants’ ankles while in a state of relaxation, using a double-echo steady-state sequence (DESS) and a 16-channel ankle coil (resolution: 0.5 × 0.5 × 0.5 mm; flip angle: 25°, repetition time: 17 ms, echo time: 6 ms) (Figure 1C) (Englander et al., 2019). Previous literature indicates that both CT and 3D MRI can be used for 2D-3D alignment. However, using MRI for alignment typically results in lower data accuracy and higher time costs. For more precise 2D-3D alignment, we utilized CT for 3D reconstruction and alignment for patients with CT images collected for clinical diagnostic needs (Akbari-Shandiz et al., 2019). For other subjects, to avoid additional radiation exposure, we used 3D MRI for alignment. The ligament attachment points were located using MRI and matched to the 3D reconstructed model from CT scans (Figure 1D).

The DBR was calibrated during each test session in order to prevent distortion and reconstruct the virtual 3D environment (Knörlein et al., 2016). Autoscoper (Brown University, United States) was used to semi-automatically track and align digitally reconstructed radiographs with synchronized biplane radiographs for each bone (Figure 1E) (Akhbari et al., 2019; Maharaj et al., 2020).

### 2.4 Calculation of ligament elongation

**FIGURE 2:**
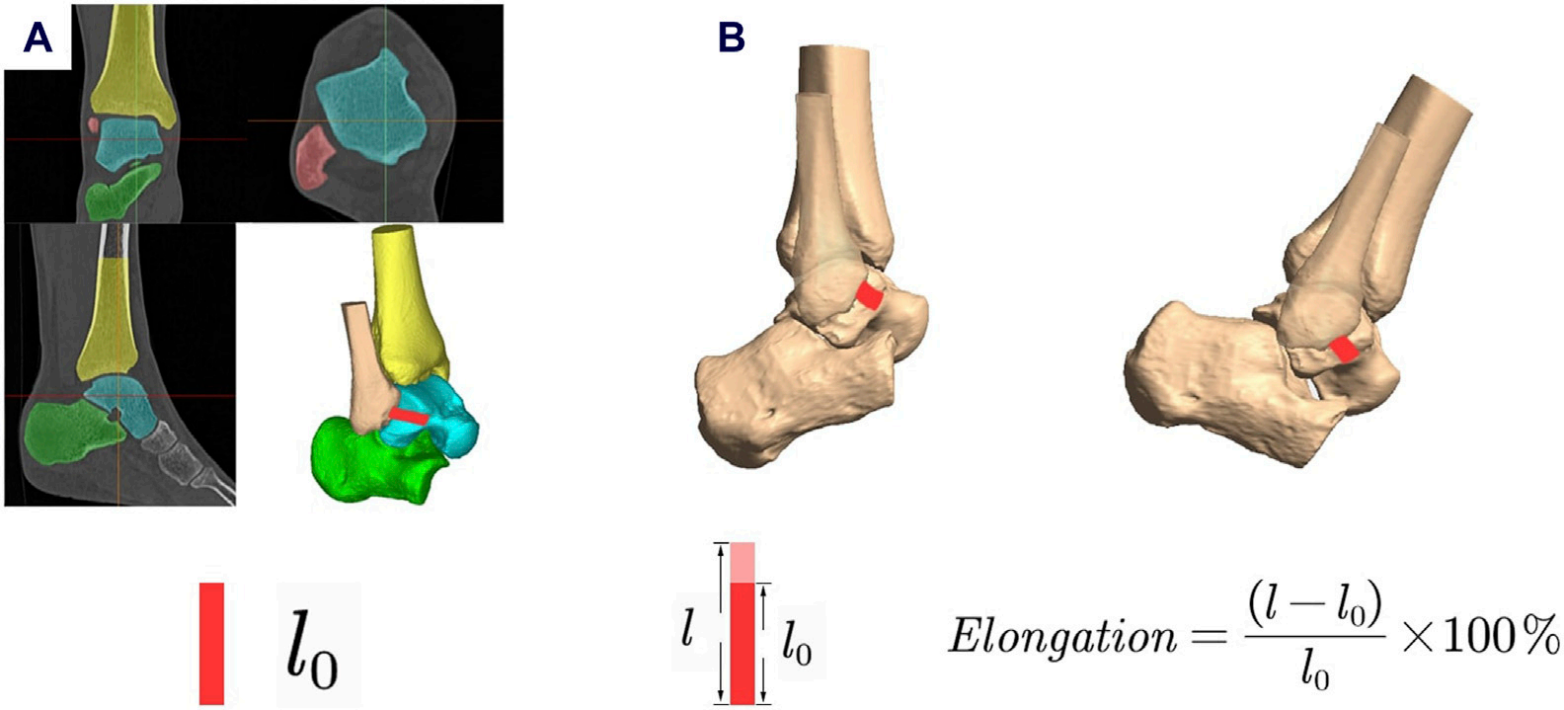
Measurement of ligament elongation. **(A)** l_0_ was determined as the length of the ligaments calculated from the MRI in its relaxed state during the MR scan; **(B)** Ligament elongation was calculated using the formula.

The study involved measuring the lengths of the ligaments by determining the shortest distances between the centroids of the attachment areas on bones. The locations and configurations of ligaments attachment sites (ATFL/CFL/PTFL) were marked in the three perpendicular imaging planes based on MRI. Ligaments elongation (%) was calculated as

$$\text{Elongation}=\frac{\left(l-l_{o}\right)}{l_{o}}\times 100\%$$

where the reference length $l_{o}$ was determined as the length of the ligaments calculated from the MRI in its relaxed state during the MR scan (Figure 2) (de Asla et al., 2009; Nukuto et al., 2023).

### 2.5 Joint kinematics calculation

The anatomical coordinate system (ACS) for each bone was determined using a combination of geometric measurements and anatomical landmarks, as outlined in previously established methodologies (Chen Wang et al., 2016; Lenz et al., 2021). The anatomical coordinate system was established as follows: For the tibia, the center of the distal articular surface was used as the origin of the coordinate system. For the talus, the center of the talus was used as the origin of the coordinate system. For the calcaneus, the center of the subtalar joint surface was used as the origin of the coordinate system. The positive directions of the axes were defined as follows: the lateral direction was defined as the positive x-axis, the anterior direction as the positive y-axis, and the proximal direction as the positive z-axis. Combining the ACS and skeletal spatial positions from DBR, we can quantify the kinematics of the tibiotalar and subtalar joints (Figure 1F). Joint angles and translations were described as relative motion of the distal bone relative to the proximal. Dynamic tibiotalar and subtalar joints angles were computed using previously established methods (Wang et al., 2023).

The foot-strike and toe-off timing were utilized to calculate the percentage of the support phase. It was noted that during the late push-off phase, the ankle and hindfoot bones were not fully captured within the view volume of the biplane radiography system for certain subjects. We retained 92% of the data from the standing phase of walking, which is the period during which all subjects were collected.

### 2.6 Statistical analysis

Variations in tibiotalar and subtalar RoM during the stance phase were detected through the utilization of paired Student’s t-tests. One-dimensional statistical parametric mapping (SPM) was employed to execute a time-domain-specific analysis of joint angles at each point of normalized percent stance. The benefit of SPM lies in its ability to conduct temporal comparisons and provide cluster-based p values (Dewig et al., 2023; Hu et al., 2023; Wattananon et al., 2023). A previous implementation of SPM (1d version 0.4, python-based open source software: www.spm1d.org) was applied (Pataky, 2012; Pataky et al., 2016). With use of paired t-tests, SPM evaluated whether kinematics and ligament elongation were statistically different between the CAI groups and contralateral, uninjured groups.

A Spearman-rho rank correlation was performed to test for a relationship between mean kinematics data (dorsiflexion, inversion, adduction, anterior, proximal, medial) and mean ligament elongation (ATFL/CFL/PTFL) for both groups. Furthermore, the relationships between ligaments elongation were quantified similarly. All statistical tests were performed in Python (3.9.1). Significance was set at *P* < 0.05 for all tests.

## 3 Results

### 3.1 Kinematics of tibiotalar and subtalar joints

**FIGURE 3:**
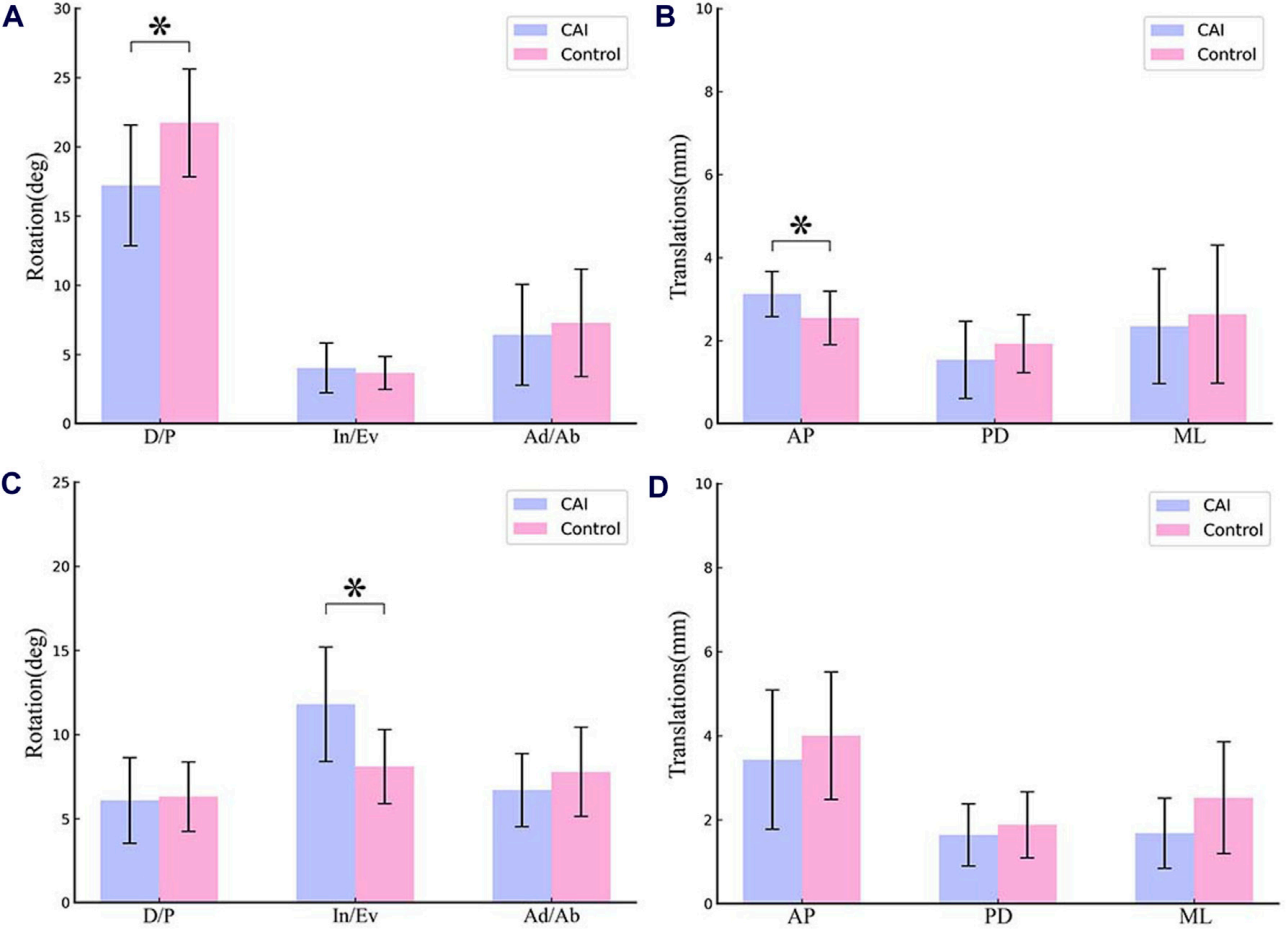
Mean RoM of the tibiotalar joint and subtalar joint during the stance phase of walking. **(A)** Rotation of tibiotalar joint; **(B)** Translation of tibiotalar joint; **(C)** Rotation of subtalar joint; **(D)** Translation of subtalar joint. (D/P, Dorsiflexion/Plantarflexion; In/Ev, Inversion/Eversion; Ad/Ab, Adduction/Abduction; AP, Anterior/Posterior; PD, Proximal/Distal; ML, Medial/Lateral).

During the stance phase, the side with CAI showed a decrease in dorsiflexion (21.73° ± 3.90° to 17.21° ± 4.35°, p = 0.01) and an increase in anterior translation of the talus (2.54 ± 0.64 mm to 3.12 ± 0.55 mm, p = 0.02) at the tibiotalar joint. Furthermore, the side affected by CAI showed an increase in inversion (8.09° ± 2.21° to 11.80° ± 3.41°, p = 0.02) at the subtalar joint (Figure 3).

**FIGURE 4:**
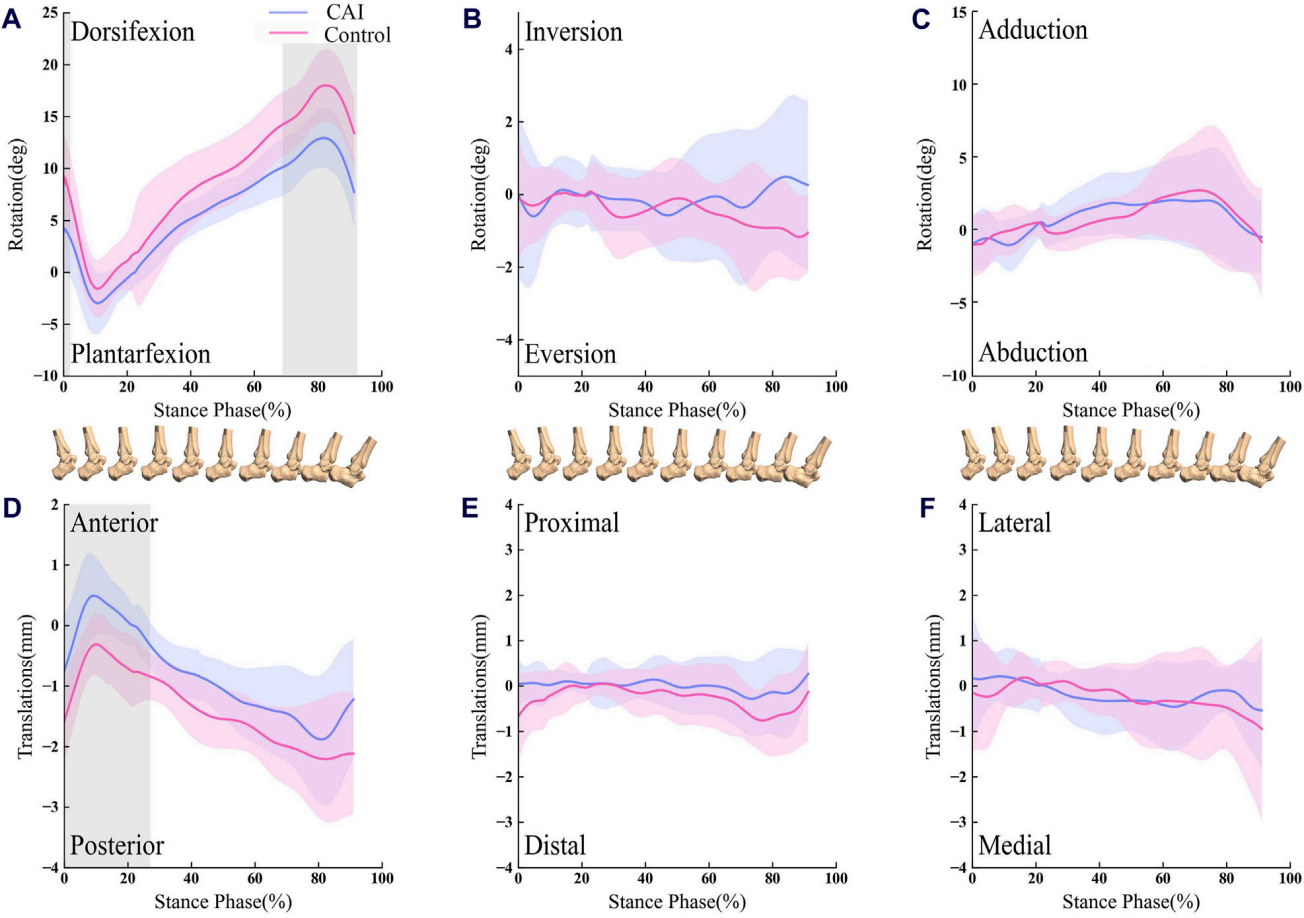
Line graph showing tibiotalar kinematics during walking for the CAI side (blue) and the contralateral, uninjured side (red). Results are normalized as the percent of the stance phase. Portions of the stance cycle during which differences were significant (in shadow) as evaluated with statistical parametric mapping are shown. The shaded regions indicate the 1SD. **(A)** Dorsifexion-Plantarfexion; **(B)** Inversion-Eversion; **(C)** Adduction-Abduction; **(D)** Anterior-Posterior; **(E)** Proximal-Distal; **(F)** Lateral-Medial.

For the tibiotalar joint, CAI ankle had a reduced dorsiflexion angle from 0% to 2.07% and 68.81%–92% of the stance phase (*p* < 0.05; maximum difference: 5.67°) (Figure 4A). The talus was more anterior relative to the tibia from 0% to 27.35% of the stance phase (*p* < 0.05; maximum difference: 0.83 mm) (Figure 4D).

**FIGURE 5:**
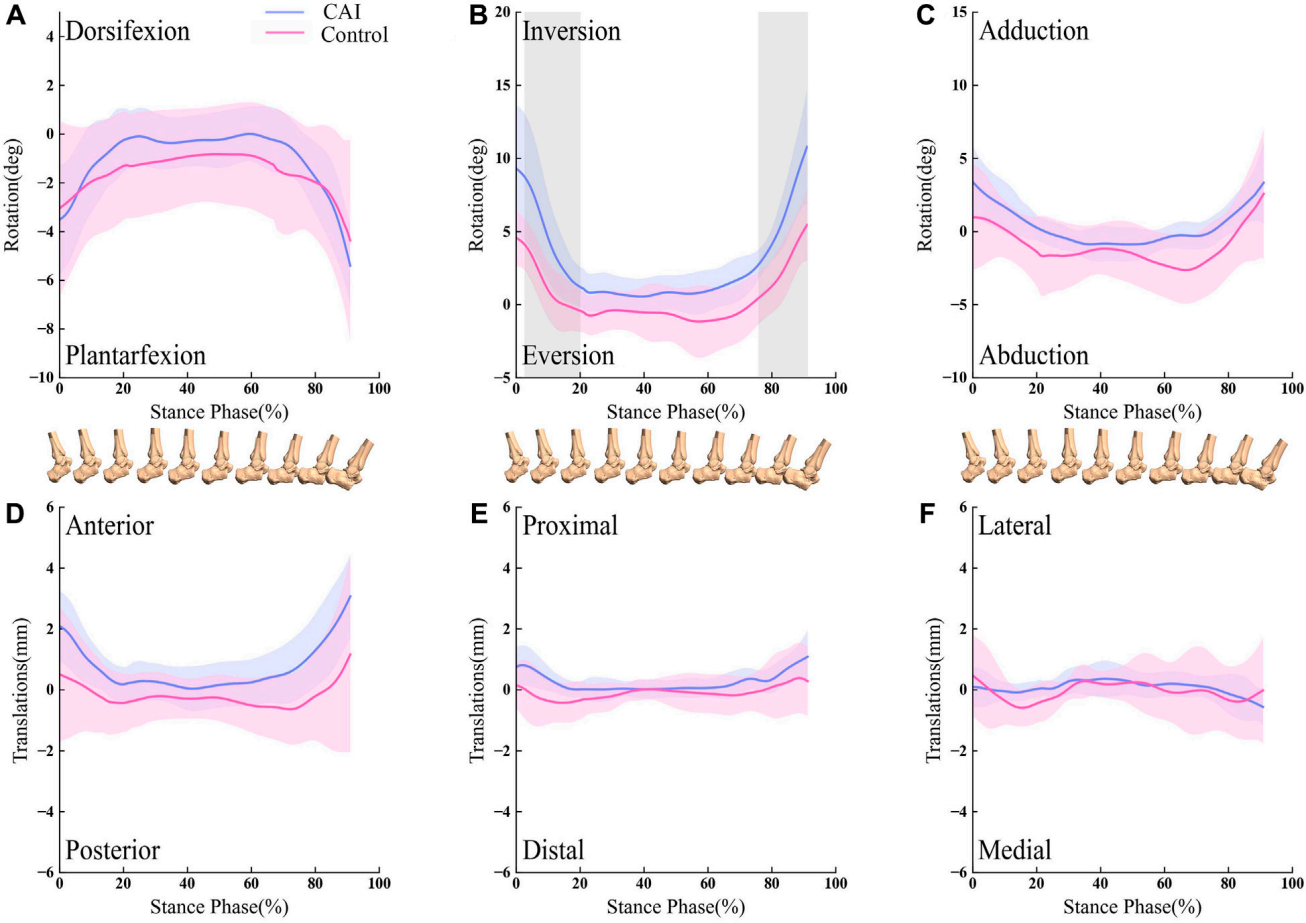
Line graph showing subtalar kinematics during walking for the CAI side (blue) and the contralateral, uninjured side (red). Results are normalized as the percent of the stance phase. Portions of the stance cycle during which differences were significant (in shadow) as evaluated with statistical parametric mapping are shown. The shaded regions indicate the 1SD. **(A)** Dorsifexion-Plantarfexion; **(B)** Inversion-Eversion; **(C)** Adduction-Abduction; **(D)** Anterior-Posterior; **(E)** Proximal-Distal; **(F)** Lateral-Medial.

For the subtalar joint, the ankle with CAI exhibited a significant increase in the inversion angle, with the phase of the stance from 2.85% to 20.24%, and 76.52%–92% (*p* < 0.05; maximum difference: 5.34°) as depicted in Figure 5B.

### 3.2 Ligaments elongation

**FIGURE 6:**
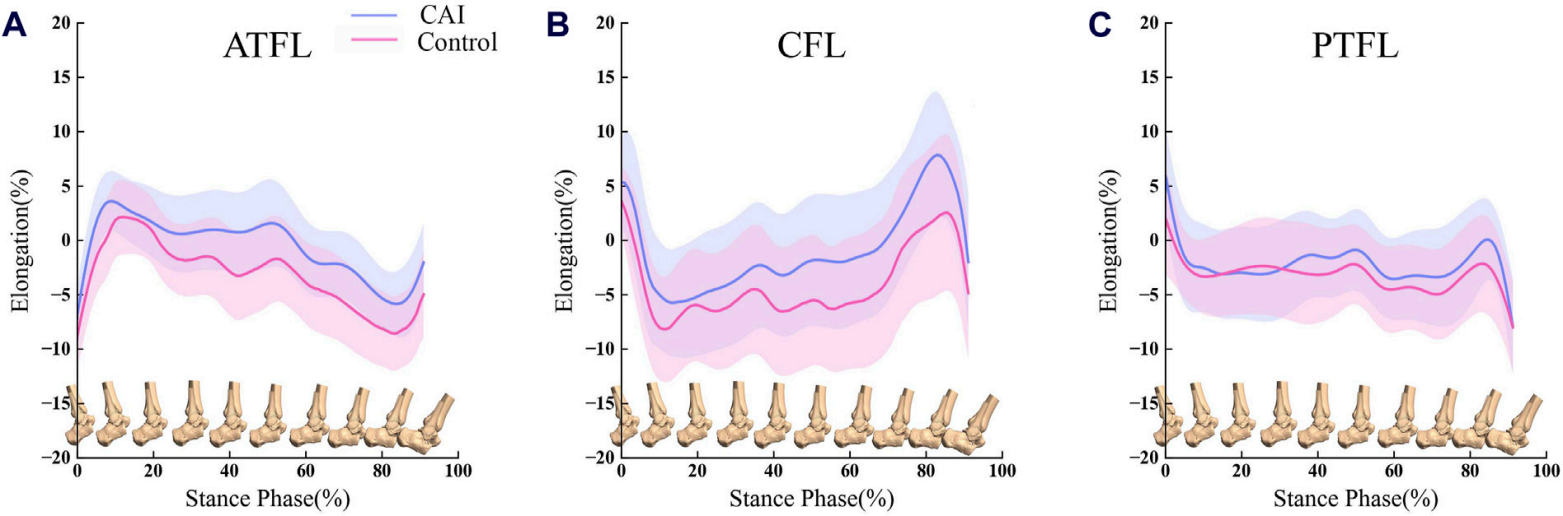
Comparison of ligament elongation between the CAI side (blue) and the contralateral, uninjured side (red). **(A)** ATFL elongation during the stance phase of walking; **(B)** CFL elongation during the stance phase of walking; **(C)** PTFL elongation during the stance phase of walking. Error bars represent 1SD.

In the stance phase, the ligaments (ATFL/CFL/PTFL) of CAI patients on the healthy and injured sides exhibit a similar tendency for elongation (Figure 6). Ankles with CAI experienced an average of 2.66% more elongation in the ATFL, 3.18% more elongation in the CFL, and 1.02% more elongation in the PTFL compared to their healthy side. However, these differences were not statistically significant.

**FIGURE 7:**
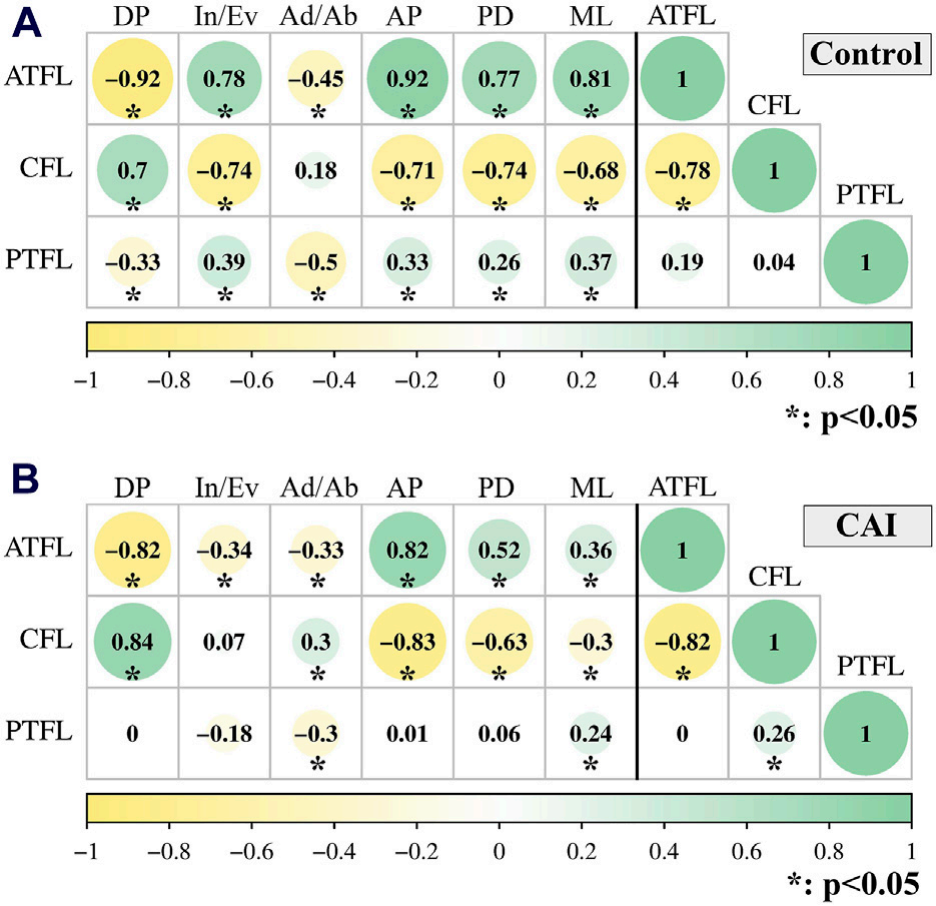
Spearman-rho correlation between joint motion (Dorsiflexion/Plantarflexion; Inversion/Eversion; Adduction/Abduction; Anterior/Posterior; Proximal/Distal; Medial/Lateral) and ligament elongation (left side of the black vertical line); and between ligaments (ATFL/CFL/PTFL) elongation (right side of the black vertical line) during walking. **(A)** uninjured group; **(B)** CAI group. The circle size, color and the number represent the rho value.

There is a dynamic correlation between tibiotalar joint motion and ligament elongation, as well as the correlation between ligaments (ATFL/CFL/PTFL) deformation (Figure 7).

**FIGURE 8:**
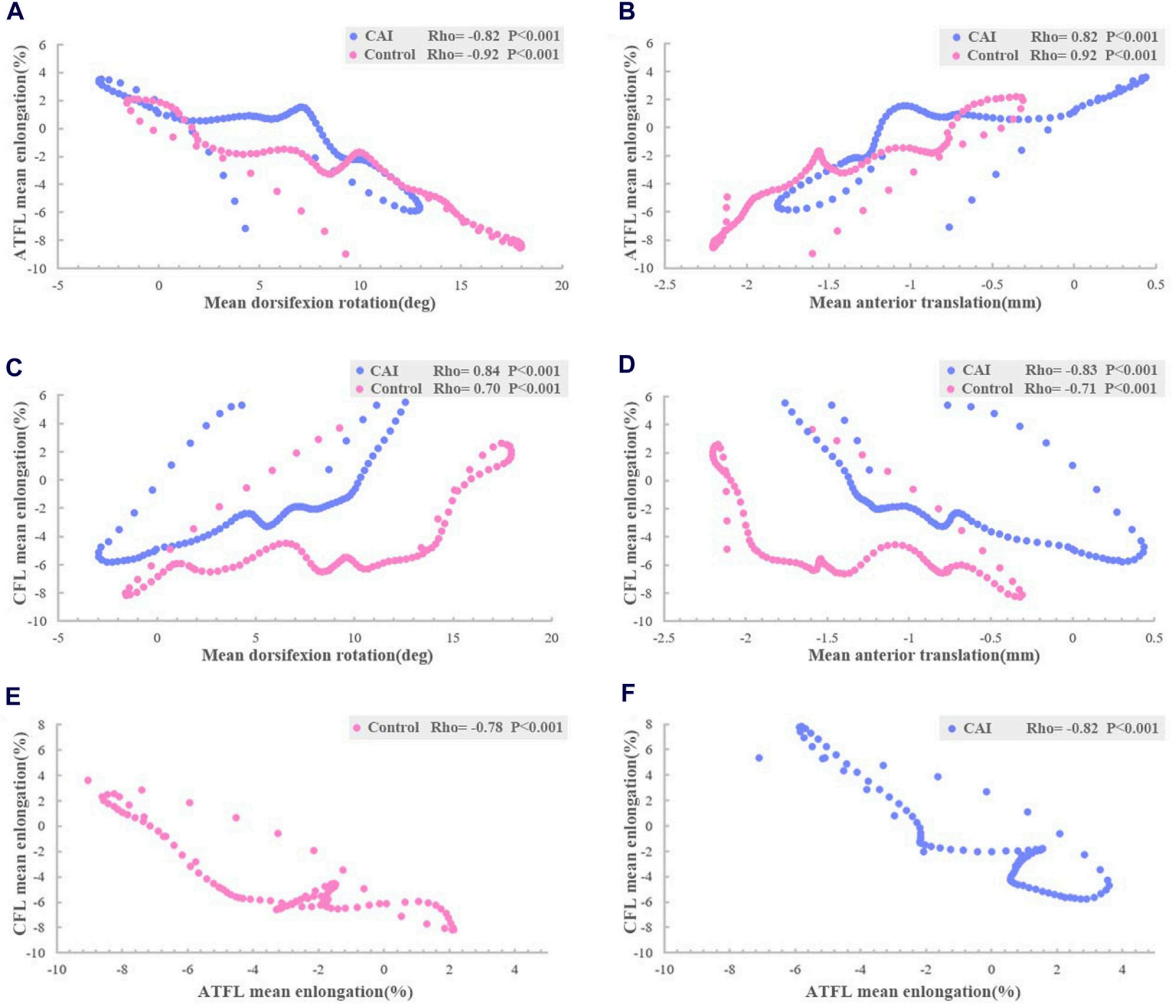
Relationship between ligaments elongation and tibiotalar kinematics during walking. Spearman-rho correlation revealed that **(A)** mean ATFL elongation was inversely related to mean dorsiflexion angle; **(B)** and related to mean anterior translation; **(C)** mean CFL elongation was related to mean dorsiflexion angle; **(D)** and inversely related to mean anterior translation; and relationship between ATFL and CTL elongation **(E)** uninjured group; **(F)** CAI group.

The relative elongation of the ATFL decreased as tibiotalar dorsiflexion (CAI: rho = −0.82, *P* < 0.001; Control: rho = −0.92, *P* < 0.001) and increased as anterior translation of talus occurred (CAI: rho = 0.82, *P* < 0.001; Control: rho = 0.92, *P* < 0.001) (Figures 8A, B). However, the relative elongation of the CFL increased as tibiotalar dorsiflexion (CAI: rho = 0.84, *P* < 0.001; Control: rho = 0.70, *P* < 0.001) and decreased with anterior translation of talus (CAI: rho = −0.83, *P* < 0.001; Control: rho = −0.71, *P* < 0.001) (Figures 8C, D).

Furthermore, the elongation of the ATFL was significantly inversely correlated with the elongation of the CFL (CAI: rho = −0.82, *P* < 0.001; Control: rho = −0.78, *P*< 0.001) (Figures 8E, F).

## 4 Discussion

In this study, we utilized DBR to calculate the *in vivo* motion of tibiotalar and subtalar joints, as well as ligament deformation in patients with CAI. This study found that there was less dorsiflexion and more anterior displacement of the talus in the tibiotalar joint, and more inversion in the subtalar joint was present on the affected side of the CAI compared to the healthy side, especially at the beginning and end of the stance phase. Furthermore, we identified a correlation between ligament elongation and tibiotalar joint motion. The elongation of the ATFL was significantly inversely correlated with the CFL, regardless of CAI. These findings may offer new insights into the motion patterns of ankle joints and connective ligaments during walking in patients with CAI.

There was less tibiotalar dorsiflexion and more subtalar inversion were found in the group with CAI. We distinguished the contribution of the tibiotalar and subtalar joints to these kinematic changes *in vivo*, although previous studies have obtained similar changes in ankle kinematics employing traditional optical motion capture methods (Tavakoli et al., 2016; Herb C. C. and Shank K., 2023; Piming et al., 2023). Inversion of the ankle mainly occurs at the subtalar joint. Previous studies have concluded that for patients with CAI, during inversion of the ankle the medial aspect of the talus likely impacts the tibial plafond, which may result in a talar osteochondral lesion and osteoarthritis (Delco et al., 2017; Yu et al., 2022; Nishimura et al., 2024). Interestingly, the present study found a significant inversion of the subtalar joint rather than the tibiotalar joint during gait. This indicates that it does not seem to provide a significant contribution to the development of ankle osteoarthritis during walking.

In this study, it was shown that kinematics differences primarily appeared during the heel strike to foot flat phase and heel off to toe off phase. It has been reported that abnormal joint kinematics are generally associated with ligament injury and muscle strength alterations in patients with CAI (Caputo et al., 2009; Herb and Hertel, 2014). Koldenhoven et al. (2016) reported that activation of the tibialis anterior in patients resulted in increased ankle plantarflexion during heel strike. We believe that the ankle joints of patients in these phases are more unstable due to ligament injury and neuromuscular abnormality, which enables them to better demonstrate the differences compared to healthy ankles.

To our knowledge, the present study is the first to conduct an *in vivo* investigation to assess the elongation of the lateral ankle ligament. This study found a high correlation between dorsiflexion angle and ATFL and CFL elongation. Numerous cadaveric studies have shown that the elongation of the ATFL increases with the plantarflexion angle, while the elongation of the CFL decreases (Ozeki et al., 2002; Takeuchi et al., 2021). However, cadaveric research cannot predict the effects of neuromuscular adaptation or dynamic loading on *in vivo* biomechanics (Canton et al., 2021). This *in vivo* evidence explains why ATFL is more vulnerable in plantarflexion, while the CFL might be more susceptible to injury in dorsiflexion.

de Asla et al. (2009) assessed the length of the lateral collateral ligament during dorsiflexion, plantarflexion, inversion, and eversion in healthy individuals using static biplane radiography. They observed a reciprocal correlation between the length of the ATFL and CFL. In this study, we refined the motion patterns of the ATFL and CFL during continuous walking measurement and found two ligaments exhibit dynamically inversely correlated elongation characteristics. A dynamic complementary relationship between these two ligaments is essential for maintaining ankle stability. Currently, ankle sagittal plane angles are not considered during ligament repair, which may affect ATFL and CFL tension. We believe that the patient’s ankle should be in a neutral position in the sagittal plane when repairing the ATFL and CFL to maintain balanced ligamentous tension.

Despite no significant differences being found, ligament elongation in the CAI group was greater than in the control group, especially in the ATFL and CFL. This may be related to the fact that the walking task may not be sufficient to reveal changes in ligament elongation in patients with CAI. In the study, only level walking was selected for the motion measurement because it is one of the most common daily activities and can be performed with low risk by CAI patients. In the future, more activities that require RoM, balance, and stability can be considered to further investigate the differences in ligament elongation in the population with CAI (Balasukumaran et al., 2020b; Canton et al., 2020). This may provide more information and insights on ankle positions that increase injury vulnerability and injury-related changes in biomechanics, therefore benefiting the design of surgical protocols.

The study had certain limitations that require further discussion. During the tracking process, it was noted that the hindfoot bones were outside the imaging range of the DBR for certain subjects at the late stage of the push-off phase. Therefore, we obtained dynamic data for 92% of the entire duration of the standing phase of the gait. This may lose valuable information at the end of the stance phase. This study focused on the lateral ligaments of the ankle *in vivo* and did not investigate the elongation of the deltoid ligament, which is also critical for ankle stability. Moreover, this study employed a convenience sample with a higher proportion of female participants due to the higher clinical incidence of CAI in females. This sampling may limit the generalizability of our findings to male patients. Future research should aim to investigate the potential kinematic and ligament elongation differences between genders with a larger and more balanced sample size.

In conclusion, patients with CAI have significant changes in joint kinematics relative to the contralateral side. Throughout the stance phase of walking, ATFL increases in length during plantarflexion and talar anterior translation whereas the elongation trend of CFL was the opposite. The interrelationship between the two ligaments is that when one ligament shortens, the other lengthens. In the future, further longitudinal research with a larger participant pool and more motion activities are necessary to determine whether the observed changes in movement and ligament stretching lead to successful postoperative outcomes.

## Data Availability

The raw data supporting the conclusions of this article will be made available by the authors, without undue reservation.
