# Supplementary material for: In vivo analysis of ankle joint kinematics and ligament deformation of chronic ankle instability patients during level walking
Source: Front Bioeng Biotechnol. 2024 Aug 6;12:1441005. doi: 10.3389/fbioe.2024.1441005 (PMC11333339; doi:10.3389/fbioe.2024.1441005)
Supplement: Supplementary file 1 [file DataSheet1.docx]

Supplementary Material

# Supplementary Tables

| **Supplementary Table1**. Demographic characteristics and radiographic grading of the degree of ligamentous injury. | | | | | | | | | |
| --- | --- | --- | --- | --- | --- | --- | --- | --- | --- |
| Case | Injured side | Sex, Age (yr) | Height (cm) | Body Mass (kg) | BMI (kg/m^2^) | ATFL | PTFL | CFL | Syndes-mosis |
| 1 | Right | F, 26 | 162 | 60 | 22.86 | III | II | III | + |
| 2 | Right | F, 36 | 171 | 75 | 25.65 | II | 0 | 0 | + |
| 3 | Left | F, 36 | 166 | 64 | 23.23 | III | 0 | II | + |
| 4 | Right | F, 54 | 160 | 75 | 29.30 | II | 0 | 0 | - |
| 5 | Right | F, 21 | 168 | 65 | 23.03 | III | 0 | II | + |
| 6 | Left | F, 26 | 168 | 56 | 19.84 | III | I | II | + |
| 7 | Right | F, 22 | 170 | 51 | 17.65 | I | 0 | II | + |
| 8 | Right | F, 33 | 158 | 60 | 24.03 | III | 0 | 0 | + |
| 9 | Left | F, 53 | 155 | 60 | 24.97 | II | 0 | I | - |
| 10 | Left | M, 53 | 180 | 84 | 25.93 | II | 0 | 0 | - |
| 11 | Left | M, 25 | 180 | 87.5 | 27.01 | II | 0 | 0 | + |
| 12 | Right | M, 35 | 175 | 70 | 22.86 | II | 0 | III | + |

# Supplementary Figure


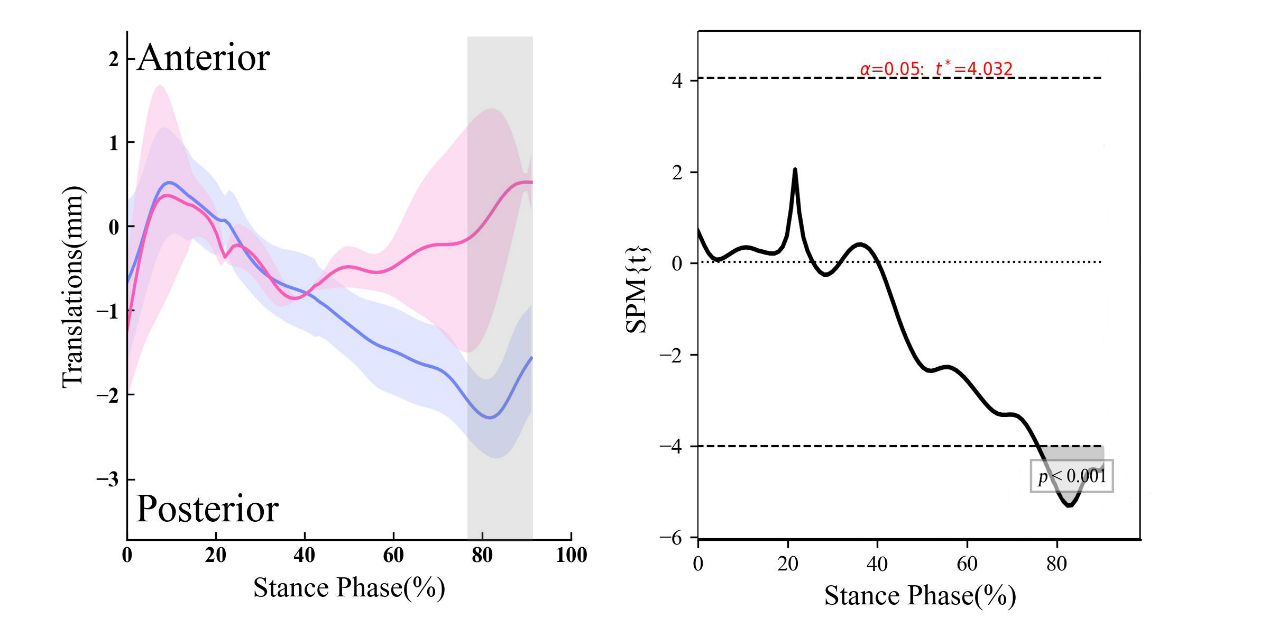


**Supplementary Figure 1.** Anterior displacement of the talus in patients with significantly severe injuries(red) versus other patients(blue).
